# Use of a Digital Assistant to Report COVID-19 Rapid Antigen Self-test Results to Health Departments in 6 US Communities

**DOI:** 10.1001/jamanetworkopen.2022.28885

**Published:** 2022-08-26

**Authors:** Carly Herbert, Qiming Shi, Vik Kheterpal, Chris Nowak, Thejas Suvarna, Basyl Durnan, Summer Schrader, Stephanie Behar, Syed Naeem, Seanan Tarrant, Ben Kalibala, Aditi Singh, Ben Gerber, Bruce Barton, Honghuang Lin, Michael Cohen-Wolkowiez, Giselle Corbie-Smith, Warren Kibbe, Juan Marquez, Jonggyu Baek, Nathaniel Hafer, Laura Gibson, Laurel O’Connor, John Broach, William Heetderks, David McManus, Apurv Soni

**Affiliations:** 1 Program in Digital Medicine, Department of Medicine, University of Massachusetts Chan Medical School, Worcester; 2 Department of Population and Quantitative Health Sciences, University of Massachusetts Chan Medical School, Worcester; 3 Center for Clinical and Translational Science, University of Massachusetts Chan Medical School, Worcester; 4 CareEvolution, Ann Arbor, Michigan; 5 Department of Pediatrics, Duke University School of Medicine, Durham, North Carolina; 6 Center for Health Equity Research, Department of Social Medicine, Department of Medicine, University of North Carolina School of Medicine, Chapel Hill; 7 Department of Biostatistics and Bioinformatics, Duke University School of Medicine, Durham, North Carolina; 8 Washtenaw County Health Department, Washtenaw, Michigan; 9 Division of Infectious Disease, Department of Medicine, University of Massachusetts Chan Medical School, Worcester; 10 Department of Emergency Medicine, University of Massachusetts Chan Medical School, Worcester; 11 National Institute of Biomedical Imaging and Bioengineering, National Institutes of Health, via contract with Kelly Services, Bethesda, Maryland; 12 Division of Cardiology, Department of Medicine, University of Massachusetts Chan Medical School, Worcester; 13 Division of Clinical Informatics, Department of Medicine, University of Massachusetts Chan Medical School, Worcester

## Abstract

**Importance:**

Widespread distribution of rapid antigen tests is integral to the US strategy to address COVID-19; however, it is estimated that few rapid antigen test results are reported to local departments of health.

**Objective:**

To characterize how often individuals in 6 communities throughout the United States used a digital assistant to log rapid antigen test results and report them to their local departments of health.

**Design, Setting, and Participants:**

This prospective cohort study is based on anonymously collected data from the beneficiaries of the Say Yes! Covid Test program, which distributed more than 3 000 000 rapid antigen tests at no cost to residents of 6 communities (Louisville, Kentucky; Indianapolis, Indiana; Fulton County, Georgia; O’ahu, Hawaii; Ann Arbor and Ypsilanti, Michigan; and Chattanooga, Tennessee) between April and October 2021. A descriptive evaluation of beneficiary use of a digital assistant for logging and reporting their rapid antigen test results was performed.

**Interventions:**

Widespread community distribution of rapid antigen tests.

**Main Outcomes and Measures:**

Number and proportion of tests logged and reported to the local department of health through the digital assistant.

**Results:**

A total of 313 000 test kits were distributed, including 178 785 test kits that were ordered using the digital assistant. Among all distributed kits, 14 398 households (4.6%) used the digital assistant, but beneficiaries reported three-quarters of their rapid antigen test results to their state public health departments (30 965 tests reported of 41 465 total test results [75.0%]). The reporting behavior varied by community and was significantly higher among communities that were incentivized for reporting test results vs those that were not incentivized or partially incentivized (90.5% [95% CI, 89.9%-91.2%] vs 70.5%; [95% CI, 70.0%-71.0%]). In all communities, positive tests were less frequently reported than negative tests (60.4% [95% CI, 58.1%-62.8%] vs 75.5% [95% CI, 75.1%-76.0%]).

**Conclusions and Relevance:**

These results suggest that application-based reporting with incentives may be associated with increased reporting of rapid tests for COVID-19. However, increasing the adoption of the digital assistant may be a critical first step.

## Key Points

**Question:**

How often do individuals use a digital assistant to log and report COVID-19 rapid antigen test results?

**Findings:**

This cohort study of 14 398 household beneficiaries of a COVID-19 test kit program in 6 US communities found that more than 75% of beneficiaries who used the digital assistant reported their rapid antigen test results to their state public health departments. Reporting behavior was significantly higher among communities that were incentivized for reporting test results.

**Meaning:**

These results suggest that application-based reporting with incentives may be associated with increased reporting of rapid tests for COVID-19.

## Teaser

This cohort study investigates the proportion of beneficiaries of a COVID-19 home test kit program who used a digital assistant to report results.

## Introduction

Rapid antigen home tests for COVID-19 are an important part of the federal government strategy to expand COVID-19 testing access and availability throughout the United States.^1^ However, the distribution and scale-up of rapid home tests for COVID-19 have been inconsistently accompanied by standard public health reporting mechanisms, challenging the ability to monitor rates of COVID-19 testing. It is important to understand more about individual reporting choices to create an optimal system for self-testing and surveillance. This study characterized how often individuals in 6 communities logged their home test results through a digital platform and patterns of result reporting state departments of health (DoH).

## Methods

This cohort study received nonresearch determination by the University of Massachusetts Chan Medical School Institutional Review Board and so was determined to be exempt from review and informed consent. The study followed the Strengthening the Reporting of Observational Studies in Epidemiology (STROBE) reporting guideline.

### Test Kit Program Intervention Communities and Procedures

The Say Yes! Covid Test program, a partnership between the National Institutes of Health and the Centers for Disease Control and Prevention, distributed more than 3 000 000 self-tests to 6 communities across the United States from April to October 2021.^2,3^ More details about the intervention can be found elsewhere.^4,5^ This analysis included data from 6 communities that finished test distribution before December 2021 and allowed users to report rapid antigen test results to the state DoH through a digital assistant: Louisville, Kentucky; Indianapolis, Indiana; Fulton County, Georgia; O’ahu, Hawaii; Ann Arbor and Ypsilanti, Michigan; and Chattanooga, Tennessee. Test kits were distributed by online ordering with direct shipment to resident homes (direct-to-consumer [DTC]) or local pick-up at community sites during the distribution periods indicated in the Table.^6^ Each household was restricted to ordering 1 test kit. In Kentucky, Indiana, Georgia, and Hawaii, test kits contained 8 rapid home tests, while kits in Michigan and Tennessee contained 25 tests.

**Table.** Users of the Digital Assistant and Tests Reported by Intervention Community

Community	Test distribution		Daily SARS-CoV-2 diagnoses/100 000 population, 7 d mean^b^	Distributed, No.		Digital DTC test kit orders, No. (%)^c^	No. (%)^a^		Test results logged per household, median (IQR)	Total tests reported to DoH, No. (%)^d^
	Start date	End date		Total kits	Tests		Users of test log	Total tests logged		
Chattanooga, TN	April 1, 2021	July 22, 2021	9.1	41 200	1 030 000	14 423 (35.0)	1366 (3.3)	2722 (0.3)	1 (1-3)	1900 (69.8)
Ann Arbor and Ypsilanti, MI	June 4, 2021	August 11, 2021	6.9	20 000	500 000	10 115 (50.6)	2382 (11.9)	8198 (1.6)	1 (1-3)	6344 (77.4)
Fulton County, GA	September 20, 2021	November 1, 2021	16.3	51 000	408 000	32 537 (63.8)	2012 (3.9)	4763 (1.2)	1 (1-3)	3742 (78.6)
O’ahu, HI	September 19, 2021	September 29, 2021	26.4	125 000	1 000 000	79 536 (63.6)	5007 (4.0)	17 140 (1.7)	2 (1-4)	11 154 (65.1)
Indianapolis, IN	October 18, 2021	November 20, 2021	19.3	35 300	282 400	22 970 (65.1)	1856 (5.3)	5724 (2.0)	2 (1-4)	5199 (90.8)
Louisville, KY	October 11, 2021	November 13, 2021	23.6	40 500	324 000	19 204 (47.4)	954 (2.4)	2918 (0.9)	2 (1-4)	2626 (90.0)
Total	NA	NA	NA	313 000	3 544 400	178 785 (57.1)	14 398 (4.6)	41 465 (1.2)	1 (1-4)	30 965 (75.0)

Abbreviations: DoH, department of health; DTC, direct to consumer; NA, not applicable.

^a^ Percentage is among total kits distributed.

^b^ Data are from the Centers for Disease Control and Prevention COVID Community Data Tracker.^6^ Mean is over the distribution period.

^c^ Percentage of total kits distributed by direct-to-consumer (DTC) orders. Remainder of kits were distributed through in-person distribution.

^d^ Percentage is among total tests logged.

### Data Collection

**Figure 1.**
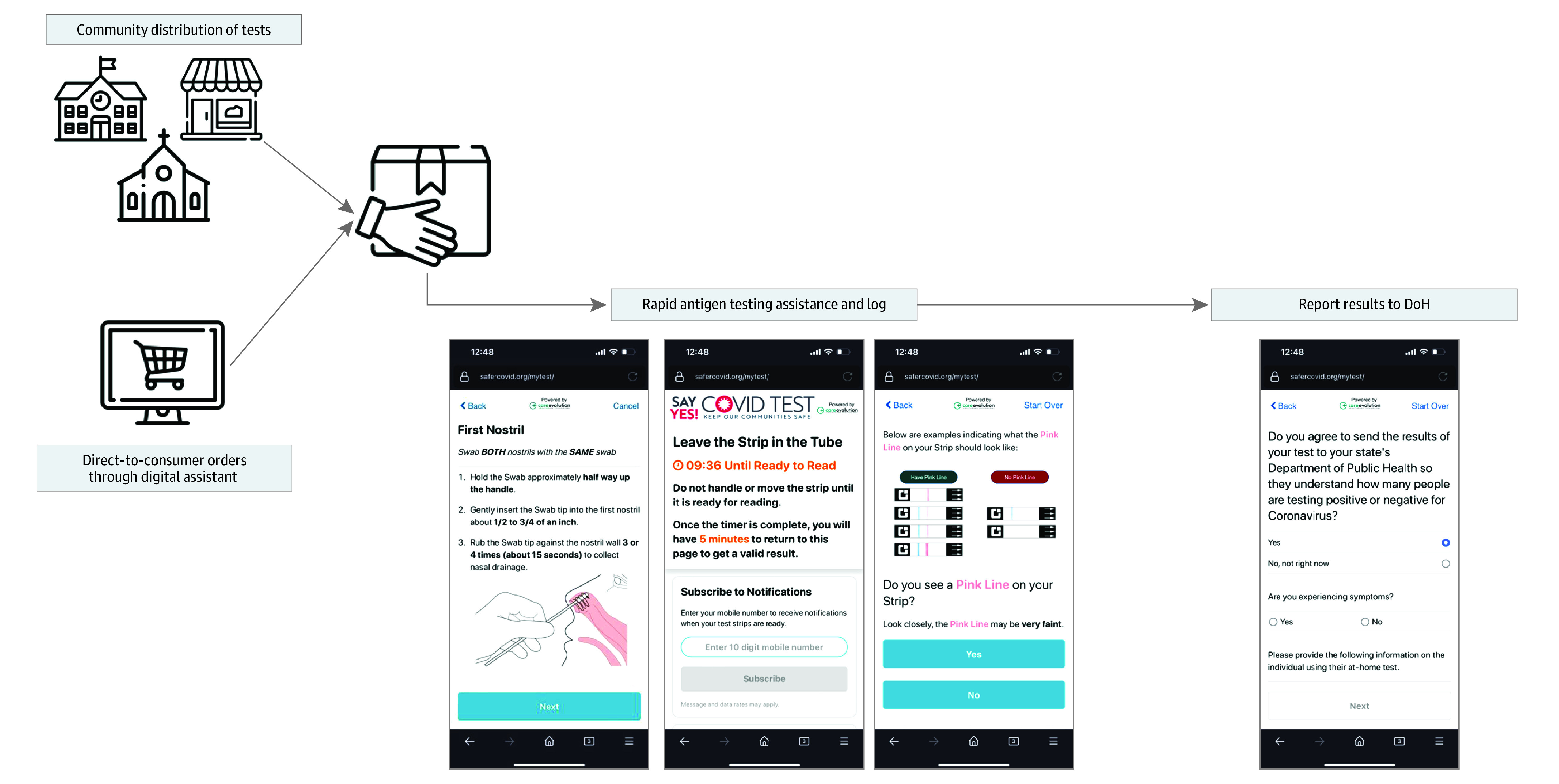
Intervention Workflow From Test Distribution to Department of Health (DoH) Reporting Screenshots are from the web-based tool.

An optional online platform and accompanying application, developed by CareEvolution, was launched with the test kit intervention as a platform for DTC orders, logging test results, and reporting results to the state DoH (Figure 1). Digital tool features were freely available and stored without personally identifiable information. Log and reporting features were available indefinitely in each site starting at the beginning of each respective distribution period. The log feature allowed households to document their test dates and results for their records. Households were also given the option to report each logged test to the state DoH through the digital assistant. For logged tests, test date, result (positive, negative, or invalid), and reporting decision (report or no report) were included in a data feed accessible to CareEvolution. For this analysis, reported tests included those reported with personally identifiable information or anonymously. A $25 gift card incentive was offered to participants in Indiana and Kentucky if they reported at least 1 test result per household to their state DoH through the digital assistant. The incentive was also offered in Georgia and Hawaii starting on October 4, and these locations were termed partially incentivized sites. No incentive was offered in Tennessee or Michigan for reporting test results to the state DoH. Beneficiaries were able to report tests at any point during and after the distribution period. Tests logged in the digital assistant from April 1, 2021, to January 12, 2022, were included in the analyses. Residents of Tennessee were unable to report tests to the DoH until June 24, 2021, so data before this point were excluded from reporting analyses for Tennessee.

### Statistical Analysis

Total numbers of DTC orders and digital assistant users were calculated by community and incentivization status (full incentive, partial incentive, and no incentive). For partially incentivized sites, the percentage of reported results was analyzed before and after the onset of incentivization. The percentage of logged tests reported to the DoH by the community with corresponding Clopper-Pearson 95% CIs were displayed graphically using R statistical software version 4.1.1 (R Project for Statistical Computing).

## Results

### Distinct Users for Logging Test Results

Of 313 000 test kits distributed through the intervention, 178 785 kits (57.1%) were ordered by households and distributed via DTC through the digital assistant, with the remainder distributed in person. Among all distributed kits, 14 398 households (4.6%) used the digital assistant (Table). COVID-19 incident rates varied by state during distribution, with the mean number of diagnoses per 100 000 residents over the prior 7 days ranging from 6.9 diagnoses in Michigan to 26.4 diagnoses in Hawaii, or 3.8-fold higher incidence. In Hawaii and Georgia, all 100 000 test kits (100%) and 34 017 of 51 000 test kits (66.7%) were distributed before the onset of incentivization, respectively. Of six intervention communities, Michigan had the greatest proportion of digital assistant users (2382 of 20 000 distributed kits [11.9%]) out of total program beneficiaries. While the median (IQR) number of tests logged by participants was 1 (1-4) test, a small number of users (415 individuals [2.9%]) logged more than 15 test results in the digital assistant.

### Reporting Behaviors

**Figure 2.**
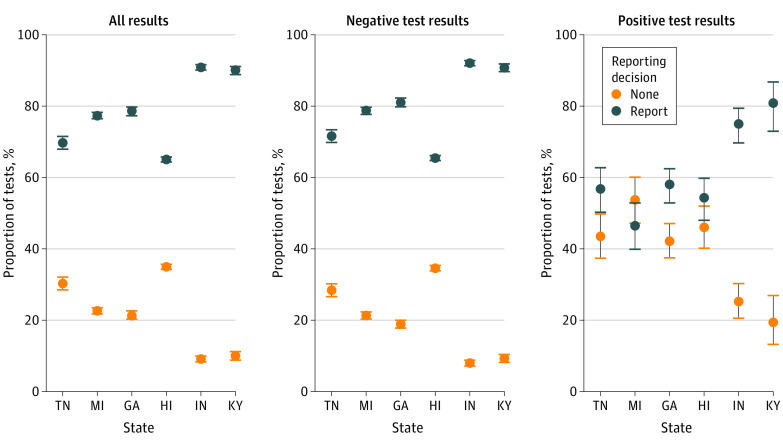
Reporting of Test Results Among Digital Assistant Users by State Reported results include those reported anonymously and with personally identifiable information. Bars indicate 95% CIs.

Three-quarters of tests logged in the digital assistant (30 965 of 41 465 total test results [75.0%]) were reported to the state DoH (Figure 2; eTable in the Supplement). Sites with complete incentivization, Indiana and Kentucky, reported a higher proportion of test results to the DoH (90.5%; 95% CI, 89.9%-91.2%) than unincentivized or partially incentivized sites (70.5% 95% CI, 70.0%-71.0%). In Hawaii, significantly more tests were reported following implementation of the incentive compared with the preincentive period (65.2% [95% CI, 64.5%-66.0%] vs 50.5% [95% CI, 43.2%-57.8%]); however, we found no difference in the reporting patterns in Georgia in the preincentive vs postincentive periods (76.2% 95% CI, 71.0%-80.9%] vs 78.7% [95% CI, 77.5%-79.9%]). The proportion of unreported results ranged from 9.2% (95% CI, 8.4%-9.9%) in Indiana and 10.0% (95% CI, 8.9%-11.2%) in Kentucky to 30.2% (95% CI, 28.5%-32.0%) in Tennessee and 34.9% (95% CI, 34.2%-35.6%) in Hawaii. In all intervention communities, positive results were less frequently reported than negative results (60.4% [95% CI, 58.1%-62.8%] vs 75.5% [95% CI, 75.1%-76.0%]). However, a higher proportion of positive results was reported from incentivized communities (76.5% [95% CI, 72.4%-80.3%]) than unincentivized (51.5% [95% CI, 47.0%-56.0%]) and partially incentivized (56.2% [95% CI, 52.5%-59.9%]) sites.

## Discussion

In this cohort study, digital assistant users who logged test results made up a small proportion (4.6%) of households who received rapid antigen tests through a home test kit initiative. However, of individuals who logged test results, approximately 75% reported their results to their state DoH. The high usage rate of the digital assistant for ordering test kits suggests that the digital assistant was accessible to intervention communities. This further suggests that low usage of the digital assistant for logging tests may be associated with inadequate community education about the importance of tracking and reporting home test results. Furthermore, there was a difference in reporting of tests by result, with positive test results significantly less frequently reported than negative results. It is important to understand and address the hesitations that may be behind reporting positive tests.

The proportion of unreported tests was nearly 3-fold higher in Tennessee and Hawaii compared with Indiana and Kentucky. This difference may be associated with differences in incentivization structures, given that participants in Indiana and Kentucky were incentivized to report tests throughout the intervention. Alternatively, test distribution in Indiana and Kentucky occurred in October 2021, after the Delta variant surge. Community awareness about the importance of reporting rapid antigen test results may have been increased at this time relative to sites with previous distribution dates.

The high proportion of application users reporting their results to the DoH in Indiana and Kentucky suggests that application-based reporting systems may be associated with an improved reporting process when paired with incentives. However, the challenge remains in drawing people to use the digital assistant, as suggested by the low uptake of the digital assistant for testing purposes. Symptom-based participatory surveillance through digital applications has been used successfully for monitoring influenza-like illness, among other infectious diseases, and rapid testing offers great opportunity to build on these technologies to rapidly ascertain changes in community prevalence of infection.^7,8^ Other means of improving uptake of the digital assistant or other reporting mechanisms should be explored further to maximize the value of these interventions.

### Limitations

This study offers a unique look into COVID-19 test reporting behaviors of nearly 15 000 digital assistant users throughout the United States. However, there are limitations to this data. The number of digital assistant users was small compared with all intervention participants, and with the current data, we were unable to assess demographics or socioeconomic status of digital assistant users or how digital assistant users compared with nonusers. Additionally, DoH reporting using the digital assistant was available to individuals who received their testes from community sites, in addition to those who used DTC ordering, and we were unable to assess whether the test distribution modality was associated with uptake of the digital assistant for logging and reporting test results. Additionally, the incidence of COVID-19 over the distribution period differed by community, with Hawaii having nearly 4-fold higher incidence of COVID-19 during the distribution period than Michigan. However, rates of COVID-19 may have changed drastically daily, weekly, or monthly, which was not reflected in these point estimates, and all sites were permitted to log and report rapid antigen tests through January 12, 2021, rather than solely during the distribution period. Further investigation is warranted to examine the association of community transmission with reporting behaviors.

## Conclusions

This cohort study found that three-quarters of individuals who used a digital assistant for testing reported their results to the DoH, suggesting that application-based reporting may be associated with increased reporting of rapid tests for COVID-19. However, the relatively low voluntary uptake of the digital assistant suggests that user-centered strategies may be necessary to maximize digital assistant usage.

## Notes

Supplement. **eTable.** Percent of Tests Reported to State Department of Health by Test Result
