# Supplementary material for: Use of a Digital Assistant to Report COVID-19 Rapid Antigen Self-test Results to Health Departments in 6 US Communities
Source: JAMA Netw Open. 2022 Aug 26;5(8):e2228885. doi: 10.1001/jamanetworkopen.2022.28885 (PMC9419013; doi:10.1001/jamanetworkopen.2022.28885)
Supplement: Supplement. — eTable. Percent of Tests Reported to State Department of Health by Test Result [file jamanetwopen-e2228885-s001.pdf]

Supplemental Online Content

Herbert C, Shi Q, Kheterpal V, et al. Use of a digital assistant to report COVID-19 rapid antigen self-test results to health departments in 6 US communities. *JAMA Netw Open*. 2022;5(8):e2228885. doi:10.1001/jamanetworkopen.2022.28885

eTable. Percent of Tests Reported to State Department of Health by Test Result

This supplemental material has been provided by the authors to give readers additional information about their work.

© 2022 Herbert C et al. *JAMA Network Open.*

eTable. Percent of Tests Reported to State Department of Health by Test Result

	All Results		Negative Test Results		Positive Test Results	
	Reported[a] % (95% CI)	Not Reported % (95% CI)	Reported % (95% CI)	Not Reported % (95% CI)	Reported % (95% CI)	Not Reported % (95% CI)
**Chattanooga, TN**	69.8 (68-71.5)	30.2 (28.5-32)	71.7 (69.8-73.4)	28.3 (26.6-30.2)	56.5 (50.2-62.7)	43.5 (37.3-49.8)
**Ann Arbor/ Ypsilanti, MI**	77.4 (76.5-78.3)	22.6 (21.7-23.5)	78.7 (77.7-79.6)	21.3 (20.4-22.3)	46.3 (39.9-52.8)	53.7 (47.2-60.1)
**Fulton County, GA**	78.6 (77.4-79.7)	21.4 (20.3-22.6)	81.1 (79.9-82.2)	18.9 (17.8-20.1)	57.8 (52.9-62.5)	42.2 (37.5-47.1)
**O'ahu, HI**	65.1 (64.3-65.8)	34.9 (34.2-35.6)	65.5 (64.7-66.1)	34.5 (33.8-35.3)	54.0 (48.0-59.8)	46.0 (40.2-52.0)
**Indianapolis, IN**	90.8 (90.1-91.6)	9.2 (8.4-9.9)	92.1 (91.3-92.8)	7.9 (7.2-8.7)	74.8 (69.7-79.4)	25.2 (20.6-30.3)
**Louisville, KY**	90.0 (88.8-91.1)	10.0 (8.9-11.2)	90.8 (89.6-91.9)	9.2 (8.1-10.4)	80.6 (73.0-86.8)	19.4 (13.2-27.0)
**Total**	75.0 (74.6-75.4)	25.0 (24.6-25.4)	75.9 (75.4-76.3)	24.1 (23.7-24.6)	60.5 (58.1-62.8)	39.6 (37.2-41.9)

[a]Reported tests include those reported with full personal identifiable information and anonymously

© 2022 Herbert C et al. *JAMA Network Open.*
